# Towards Addressing COVID‐19 Vaccine Wastage in Cameroon: Challenges and Way Forward

**DOI:** 10.1002/puh2.200

**Published:** 2024-06-21

**Authors:** Nathan Ezie Kengo, Fodop Samuel Ghislain, Yvan Zolo, Shuaibu Saidu Musa, Mukhtar Musa Shallangwa, Usman Abubakar Haruna, Emery Manirambona, Don Eliseo Lucero‐Prisno

**Affiliations:** ^1^ Faculty of Medicine and Biomedical Sciences of Garoua University of Garoua Garoua Cameroon; ^2^ Winners Research Foundation Yaounde Cameroon; ^3^ School of Health and Medical Sciences Catholic University of Cameroon Bamenda Cameroon; ^4^ Global Surgery Division University of Cape Town Cape Town South Africa; ^5^ Department of Nursing Science Ahmadu Bello University Zaria Nigeria; ^6^ Achieving Health Nigeria Initiative Biu Nigeria; ^7^ Department of Biomedical Sciences Nazarbayev University School of Medicine Astana Kazakhstan; ^8^ College of Medicine and Health Sciences University of Rwanda Kigali Rwanda; ^9^ Department of Global Health and Development London School of Hygiene and Tropical Medicine London UK

**Keywords:** Cameroon, COVID‐19, vaccine wastage, way forward

## Abstract

The coronavirus‐2019 (COVID‐19) pandemic has tremendously impacted both the small and large world economies. Schools and businesses were shut down and national borders closed, interrupting international trade and movement of people. This eventually led to huge economic losses and rendered many people jobless. Unlike in high‐income countries, recovery in the post‐pandemic period is yet to be fully actualized as many countries, especially from Africa that were hit the most by the pandemic continue to face other health and economic setbacks. The African continent is endemic to diseases such as tuberculosis, malaria and HIV which already required funding before the COVID‐19 pandemic. During the pandemic, the Cameroonian health authorities reported numerous confirmed cases. Vaccination continues to be an effective means to avoid further spread of the virus and minimize possible occurrences of other COVID‐19 variants across the globe. Despite the efforts made towards COVID‐19 vaccination, only 12% of Cameroonians were reported to have completed the COVID‐19 vaccinations in the basic series. In the country, multiple resorts were used to attenuate the impact of the virus, including barrier measures, vaccines and even traditional therapies. The initial promotion of traditional remedies as control measures created a preference over vaccines due to misinformation from social media platforms, contributing to fear of vaccines, and consequently resulted in a high vaccine hesitancy (VH) reported at 56.9%. This VH coupled with cold chain management challenges resulted in vaccine wastage. Consequently, the target of vaccinating 15 million Cameroonians by December 2022 to reach the threshold vaccination coverage expected to confer immunity was not attained. Hence, it is important to reduce expenditures on extra doses of vaccines, maximize uptake through vaccination sensitization campaigns and increase access to avoid vaccine wastage. This will be instrumental in attaining herd immunity and contribute to the fight against new COVID‐19 variants.

## 1 Introduction

It is over 4 years since the coronavirus‐2019 (COVID‐19) pandemic. The post‐pandemic period is characterized by several measures put in place by both international and national organizations to avoid new cases, to better manage existing cases and coinfections and to avoid the occurrence of new variants of the virus. As of 22 January 2024, a total of 774,075,242 COVID‐19 cases were reported globally [1]. Of these cases, 9568,429 (1.2%) were reported from Africa. Among these, Cameroon accounted for 125,109 confirmed cases with a total of 1974 deaths; representing a case fatality rate of 1.6% [1]. Isolation of suspected cases, frequent hand washing, social distancing and the use of personal protective equipment were among the measures taken to curb the spread of the virus. Following scientific studies and recommendations, the utilization of vaccines for preventing COVID‐19 transmission was equally introduced with the approval of the *Pfizer‐BioNTech COVID‐19 Vaccine* by the US Food and Drug Administration on 23 August 2021. The vaccine has been available since 11 December 2020, under emergency use authorization [2]. However, the degrees of implementation of these preventive measures were not the same as the different world economies were not hit the same way. Therefore, some countries mandated vaccination for their citizens, whereas others did not. Consequently, recovery in the post‐pandemic period is even more challenging for low and middle‐income countries such as Cameroon which are already faced with socio‐political instabilities and endemicity of diseases such as malaria, cholera and tuberculosis, among others. Amidst these is the likelihood of COVID‐19 vaccine wastage due to poor cold chain system and vaccine hesitancy (VH), even though immunization has proven to be an effective measure to fight the COVID‐19 pandemic and the emergence of its variants.

## 2 Covid‐19 Vaccine Utilization in Cameroon

Following recommendations from the World Health Organization (WHO), the Cameroonian health authorities started COVID‐19 vaccination on 12 April 2021, for high‐risk groups [3] such as healthcare professionals and the aged. To boost vaccination uptake, the government created 243 vaccination sites across the national territory for its population of over 28 million inhabitants. The vaccines in Cameroon were donated by other countries such as China, which donated one million doses of its Sinopharm vaccine. Japan also donated approximately 70 000 doses of COVID‐19 vaccines on 17 April 2022 [4, 5]. The United States of America also donated 847 310 doses of the vaccines to the Cameroonian government. These included 208 260 Pfizer and 639 050 J&J COVID‐19 vaccines. The donations were received through the COVID‐19 Vaccines Global Access facility to help the country in the fight against COVID‐19 [6] and prevent the emergence and spread of its variants.

As of 22 July 2023, a total of 4792,066 vaccine doses had been administered in Cameroon [1]. This figure represents only 6.8% of the entire Cameroonian population which is ranked 52nd in the list of countries by population around the globe. The country's population currently stands at approximately 28,129,186 inhabitants [7].

It is worth noting that only about 12% of Cameroonian citizens had gotten the complete COVID‐19 vaccinations in the basic series, whereas only 3% had received one booster dose of a COVID‐19 vaccine as of 26 November 2023 [1]. The unused vaccines were consequently exposed to expiration and eventually wasted. This is accounted for by a high rate of VH estimated at 56.9% among healthcare workers for instance; a phenomenon known as ‘a delay in acceptance or refusal of vaccines despite availability of vaccination services’ [8]. As a result, about 391,200 doses of the Covishield vaccines in Cameroon were reported expired in August 2021 [3]. This will therefore threaten the country's efforts to fight the emergence of COVID‐19 variants and their sub‐variants.

According to the country's projections, the country aimed to vaccinate 5400,000 people against COVID‐19 by 2021, followed by 15 million more by December 2022 in an effort to reach the vaccination coverage threshold (to vaccinate 40% of the total population) expected to confer immunity [3]. However, this was not realized, and efforts made by Cameroon to curb the virus are in jeopardy.

## 3 Challenges and Causes of Vaccine Wastage in Cameroon

Currently, the projected COVID‐19 vaccination coverage in Cameroon is far from reality. This is partly a result of VH attributed to the promotion and consumption of traditional herbal remedies to control COVID‐19. At the start of the crisis, the Cameroonian government officially authorized the marketing of four improved traditional medicines to fight against COVID‐19 [9, 10]. These traditional medicines were massively consumed as preventive and treatment modalities before the international legislation of the COVID‐19 vaccine. Another contributing factor to the VH was misinformation from social media. Upon the institution of the vaccine, some people were of the opinion that the COVID‐19 vaccine is detrimental to the health of persons of colour [11]. The lack of evidence‐based information discouraged citizens from taking the first dose or returning for the subsequent doses [11]. As a result, the majority of vaccines administered in Cameroon were limited to a few categories of people, including strangers, Cameroonians who require the vaccine for traveling purposes, and those obliged by their employers to be vaccinated [11]. Therefore, slews of the vaccines were wasted as a large number of Cameroonians remain unvaccinated for fear of the unknown.

Poor cold chain system and vaccine accessibility are other challenges resulting in vaccine wastage. The available vaccination sites are either insufficient or inaccessible due to poor road networks [12]. This has further discouraged the already financially crippled citizens to receive the vaccine. The perpetual political instability in the country is another threat to the vaccination campaign in Cameroon. The imposed ghost towns and risk zones in the country's Northwest and Southwest Regions have negatively impacted the vaccination progress in the country's English‐speaking areas where the movements of people are restricted. Moreover, the unavailability of Ultra Cold (−90 to −60°C) conditions for vaccine storage and usage before 12 months to expiration date after manufacture signifies that most vaccines may not be utilized at the current uptake rate [12]. The poor cold chain system and the variability of temperature in the different regions of Cameroon pose a risk to the stability or potency of these vaccines. This hinders vaccine security, which is the timely, continuous, uninterrupted supply of reasonably priced vaccines with guaranteed quality [13]. The insufficiency of such accessibility, storage and management conditions poses significant threats to achieving COVID‐19 herd immunity in Cameroon.

It, therefore, becomes a huge burden for this global south country to curb the emergence of COVID‐19 variants and potential co‐infections.

## 4 Efforts in Preventing Vaccine Wastage in Cameroon

Regarding measures put in place to limit vaccine wastage, the Cameroonian authorities partnered with several organizations. Amongst these, is the Ministry of Public Health's partnership with the WHO, UNICEF and religious associations to sensitive and encourage community involvement regarding COVID‐19 vaccination as a means of combating the coronavirus [14] and its emerging variants and sub‐variants.

Since the start of the pandemic, the Cameroonian government has organized five COVID‐19 vaccination campaigns for people aged 18 and above. The fifth, which was officially launched by the Minister of Public Health, started on 18 November 2022 [15]. During these campaigns, the elderly, people with conditions like diabetes or high blood pressure, and medical professionals were given priority.

During the last vaccination campaign, three vaccinations were made available to the Cameroonian population: Sinopharm, Pfizer and Johnson & Johnson in two doses separated by 21 days. Multiple sites, including health institutions, public areas (markets, chifferies, churches and mosques, administrative buildings, enterprises) and other meeting spots, determined as part of operational planning were all used for vaccinations [15]. According to the health ministry, these national vaccination campaigns were employed to intensify vaccine uptake and achieve collective and robust immunity.

## 5 Conclusion and Recommendations

Managing a pandemic such as the COVID‐19 that crippled many economies and health systems around the world can be challenging especially for a country with an overburdened and fragile healthcare system like Cameroon. Therefore, the timely and effective use of the already available vaccines should be prioritized. Misinformation about COVID‐19 and its vaccination should be properly addressed to encourage vaccine uptake. The cold chain system should be adequately equipped to ensure vaccine safety and efficacy. Moreover, security should be provided in the conflict‐affected regions for the safety of healthcare professionals and the people who are willing to contribute to herd immunity by getting vaccinated. Good road networks and well‐functioning vehicles should be provided for the smooth and timely transport of vaccines and healthcare professionals, especially in hard‐to‐reach areas. This will help Cameroon to curb the spread of COVID‐19 and prevent the emergence of its variants. Moreover, these measures can contribute to better health outcomes of citizens and foster Cameron's healthcare resilience and recovery after the pandemic and beyond.

## Author Contributions

All listed authors contributed significantly to this work. They all approve the final version of the article for publication.

## Conflicts of Interest

Shuaibu Saidu Musa, Usman Abubakar Haruna and Emery Manirambona are Youth Editorial Board members of Public Health Challenges and Don Eliseo Lucero‐Prisno is the Chief Editor of the journal. They are co‐authors of this article. To minimize bias, they were excluded from the peer‐review process and all editorial decision‐making related to the acceptance of this article for publication.

## Ethical Statement

None.

## Data Availability

No new data were created or analysed during this study. Data sharing is not applicable to this article.
